# Workplace stress and associated factors among healthcare professionals working in public health care facilities in Bahir Dar City, Northwest Ethiopia, 2017

**DOI:** 10.1186/s13104-019-4277-1

**Published:** 2019-05-02

**Authors:** Selam Gebeyehu, Balew Zeleke

**Affiliations:** 1 Amhara Metal Industry and Machine Technology Development Enterprise, Bahir Dar, Ethiopia; 2 School of Nursing, College of Medicine and Health Science, Bahir Dar University, Bahir Dar, Ethiopia

**Keywords:** Workplace stress, Health care professionals, Health care facilities, Bahir Dar, Ethiopia

## Abstract

**Objective:**

The objective of this study was to assess workplace stress and associated factors among health care professionals working in public health care facilities in Bahir Dar city, Northwest Ethiopia, 2017.

**Results:**

Out of the expected 294 study participants, 253 respondents had participated in the study making the response rate 86.1%. In this study the prevalence of workplace stress was found to be 48.6%. Educational status (AOR: 3.227, 95% CI [(1.358, 7.673) and working experience (AOR: 2.11,95, 95% CI [1.046, 4.260]) were the factors associated with workplace stress. The current study concluded that the prevalence of workplace stress was high among the study participants. Therefore, this study recommended that the health care facilities should work to identify other sources of workplace stress and further large-scale researches need to be done.

## Introduction

The word “stress” was defined by different scholars. The word was first defined by Hans Selye in 1936 as “a non-specific response of the body to any demand of change”. Workplace stress occurs in all workers or employees and high among health-care professionals an important group that can be affected by workplace stress because of the nature of their work environment [1]. Workplace stress can have a number of impacts on health including mental and behavioral disorders such as exhaustion, burnout, anxiety and depression, as well as other physical impairments such as cardio-vascular disease and musculoskeletal disorders [2]. In addition, stress can result in work absences, higher turnover, early retirement, lower productivity, and lower quality of services or products [3]. Previous studies revealed that there were different factors which contribute for workplace stress. For instance, the study conducted in Addis Ababa revealed that gender, work shift, illness, marital status, and work unit were significantly associated with workplace stress and [4]. The study done in Kampala Uganda also showed that work experience and number of children were significant risk factors of workplace stress [5]. Another study done in Melbourne, Australia stated that determinants of workplace stress were social support, nurse empowerment and self-determination [6]. Similarly, a study done in Jordan revealed that workplace stress was highest among general practitioners, dentists, pharmacists and lowest among physician specialists [7]. A study done in New Zealand showed that excessive paper work, health reforms, bureaucratic interference, excessive hours and on-call work were the major factors associated with workplace stress [8]. A study conducted in Worabe, South West Ethiopia, pointed out that sex, age, religion, ethnicity, marital status, child rearing, professional qualification, monthly salary, work experiences and department of work were the contributing factor of workplace stress [9].

Similarly, a study done in Iran concluded that the risk factors of workplace stress were gender, hospital ward and working shift [10].

Almost all of the above researchers had examined workplace stress and associated factors among a single health care professional group. This leads us to dig out workplace stress and associated factors across different health care professionals. Thus, conducting of this research would contribute evidences to the existing literature in the area of workplace stress. Therefore, the objective of this study was to assess workplace stress and associated factors among health care professionals working in public health care facilities in Bahir Dar City.

## Main text

### Methods

#### Study design, area and period

An institution based cross sectional study design was conducted among health care professionals working in public health care facilities in Bahir Dar city, Northwest Ethiopia. The study was conducted from March 1st–April 30, 2017.

Bahir Dar is the capital city of Amhara National Regional State located 563 km away from Addis Ababa, the capital city of Ethiopia. In the city there were eight public health facilities; two hospitals (Felege Hiwot Hospital and Addis Alem Hospital) and six health centers (Abay Health center, Han health center, Shimbet health center, Ginbot 20 health center, Bahir Dar health center and Shumabo health center). There were 803 health care professionals working in all of the above listed public health care facilities [11].

#### Population

The source population of the study were all health care professionals who were working in the public health care facilities and the study population were all health care professionals who were working in the selected health care facilities at the time of data collection.

#### Inclusion and exclusion criteria

All health care professionals who were working 6 months and above were included in the study whereas all healthcare professionals who were working less than 6 months and on annual leave were excluded from the study.

#### Sample size determination and sampling technique

##### Sample size determination

Since the number of population size under study is finite/known, the researchers were using the following simplified formula”

$$N/1 + N(e)^{2}$$

where, n = sample size, N = size of the population; 803, e = level of precision; 0.05.

Thus 803/1 + (803) (0.05)^2^ and adding 10% non-response rate, the total sample size was 294.

##### Sampling technique

Two hospitals and two health centers were selected by simple random sampling technique. The calculated sample size was allocated proportionally to each health care facilities based on the number of populations. List of health care professionals was obtained from Human resource (HR) departments of each health care facilities. Finally, simple random sampling method was used to approach the study participants.

#### Study variables

##### Dependent variable

Workplace stress.

##### Independent variables

Socio-demographic characteristics (sex, age, profession, marital status, income, working experience, ethnicity and level of education), demand factors (work load, work pattern, work environment and work content), relationship factors (relationship with colleagues, relationship with managers and work ethics), and support factors (encouragement, sponsorship, financial support and incentives).

#### Data collection instrument

Data were collected by pretested and structured self-administered questionnaire developed through adaptation from Management Standards’ and work-related stress in the UK developed by Rosanna Cousins et al. [12]. The questionnaire has four parts socio-demographic characteristics, job demand, work relationship and managerial support. Job demand has eight items, managerial support has five items and work relationship has four items. A five-type Likert scale was used. Responses were rated as either 1 (never), 2 (seldom), 3 (sometimes), 4 (often), 5 (always) for all sub scales. Only the last three were qualified as stressed. The scores range from a minimum of 17 to a maximum of 85. The higher the score the more stressed the health care professionals. Percentages mean score of work-related stress was calculated by the following formula [13].

$${\text{Percentages mean score of work-related stress}} = \frac{{\text{Actual computed mean score}}}{{\text{Maximum potential score}}} \, \times 100$$

#### Data quality control

Two days training was given to the data collectors. Data were collected by six-degree nurses who were working outside the selected hospitals. The data collectors were supervised daily by the supervisors. The questionnaire was pretested on 5% of the calculated sample size among health care professionals working in a nearby Hospital called “Merawi” Hospital. Modifications were made based on pretest data analysis.

#### Data processing and analysis

The collected data were edited and entered into Epi data version 3.1 and were exported into SPSS version 23 for analysis. All variables assessed in the model were categorical variables. The statistical association between dependent variable (workplace stress) and independent variables [Socio-demographic characteristics (sex, age, profession, marital status, income, working experience, ethnicity and level of education), demand factors (work load, work pattern, work environment and work content), relationship factors (relationship with colleagues, relationship with managers and work ethics), and support factors (encouragement, sponsorship, financial support and incentives)] was assessed using logistic regression model. All variables with p-value of < 0.2 in bivariable logistic regression model were entered into multivariable logistic regression model. Finally, a p value < 0.05 in multivariable model was set as statistically significant.

### Results

Out of 294 study participants, 253 were participating in the study making the response rate 86.1%. The majority, 199 (78.5%) of the respondents were working from Hospital. One hundred thirty-eight (54.5%) and 93 (36.8%) of the respondents were male and in the age range of 25–29 years old respectively. The majority, 145 (57.3%) and 154 (60.9%) were married and bachelor’s degree respectively. Two hundred thirty-five (92.9%) of the respondents were from the Amhara Ethnicity and the majority, 102 (40.3%) of the respondents were nurses by profession. About 110 (43.5%) of the respondents had greater than 5 years working experiences and almost half of 125 (49.4%) of the respondents had earned less than 174.22 USD per month (Table 1).

**Table 1:** Socio-demographic characteristics among health care professionals working in public Health care facilities of Bahir Dar city North West Ethiopia, 2017

Variables	Category	Frequency	(%)
Working health facility	Hospital	199	78.7
	Health center	54	21.3
Sex	Male	138	54.5
	Female	115	45.5
Age	Up to 24 years	20	7.9
	25–29 years	93	36.8
	30–34 years	83	32.8
	≥ 35 years	57	22.5
Marital status	Married	126	49.8
	Single	127	50.2
Educational status	Bachelor’s degree	154	60.9
	Diploma	63	24.9
	Master’s degree	36	14.2
Ethnicity	Amhara	235	92.9
	Oromo	12	4.7
	Others	6	2.4
Profession/job category	Physicians	100	39.5
	Nurses/midwifery	124	49.0
	Others	29	11.5
Working experience	½–5 years work experience	227	89.7
	> 5 years work experience	110	43.5
Monthly income	< 174.22 USD	125	49.4
	174.22–348.44 USD	113	44.7
	> 348.44 USD	15	5.9

#### Factors associated with workplace stress

Multivariable logistic regression indicated that educational status and working experience were found to be associated with workplace stress Respondents who were Master’s degree were three times (AOR: 3.227, 95% CI [(1.358, 7.673]) more likely to experience workplace stress. Similarly, participants who had worked greater than 5 years were two times (AOR: 2.11, 95% CI [1.046, 4.260]) more likely to experience workplace stress than respondents who had worked ½–5 years (Table 2).

**Table 2:** Factors associated with work place stress among health care professionals working in public health care facilities in Bahir Dar City, Bahir Dar, Ethiopia, 2017

Variable	Response	Work place stress		COR (95% CI)	AOR (95% CI)
		Yes (%)	No (%)		
Working health care facility	Hospital	94 (47.2)	105 (52.8)	0.772 (0.42, 1.41)	
	Health center	29 (53.7)	25 (46.3)	1	
Sex	Male	62 (44.9)	76 (55.1)	1	
	Female	61 (53.0)	54 (47.0)	0.722 (0.44, 1.19)	
Age	Up to 24 years	12 (60.0)	8 (40.0)	3.00 (1.05, 8.58)*	1.51 (0.45, 5.020)
	25–29 years	50 (53.8)	43 (46.2)	2.33 (1.17, 4.61)*	1.84 (0.87, 3.89)
	30–34 years	42 (50.6)	41 (49.4)	2.05 (1.02, 4.12)*	2.046 (0.97, 4.31)
	≥ 35 years	19 (33.3)	38 (66.7)	1	1
Marital status	Married	57 (45.2)	69 (54.8)	1	
	Single	66 (52.0)	61 (48.0)	1.310 (0.80, 2.15)	
Educational status	Bachelor’s degree	63 (40.9)	91 (59.1)	1	1
	Diploma	34 (54.0)	29 (46.0)	1.693 (0.938, 3.056)	1.703 (0.904, 3.208)
	Master’s degree	26 (72.2)	10 (27.8)	3.756 (1.693, 8.332)*	3.227 (1.358, 7.673)*
Profession/job category	Physicians	49 (49.0)	51 (51.0)	0.752 (0.442, 1.277)	1
	Nurses/midwifery	52 (41.9)	72 (58.1)	3.271 (1.282, 8.345)*	0.794 (0.431, 1.460)
	Others	22 (75.9)	7 (24.1)	1	2.499 (0.931, 6.707)
Working experience	½–5 years	37 (64.9)	20 (35.1)	2.37 (1.28, 4.37)*	2.11 (1.046, 4.260)*
	> 5 years	86 (43.9)	110 (56.1)	1	1
Monthly income	< 174.22 USD	55 (57.3)	41 (42.7)	1.23 (0.60,2.51)	
	174.22–348.44 USD	45 (39.8)	68 (60.2)	0.60 (0.30, 1.22)	
	> 348.44 USD	23 (52.3)	21 (47.7)	1	

* Statistically significant at p < 0.05

### Discussion

The current study was conducted to assess workplace stress and associated factors among healthcare professionals working in public health care facilities in Bahir Dar city, Northwest Ethiopia. The prevalence of workplace stress in this study was found to be 48.6%. This finding is consistent with the previous study done in West Sussex (43%) [14] and Mekelle (46.9%) [15].

The current finding is lower than the previous studies done in Botswana (74%) [16], Saudi Arabia (66.2%) [17], India (73.5%) [18] and Dutch (55%) [19]. The reason for this difference might be due to the sample size (the smaller sample size in the current study) and the study settings differences. The current study finding is higher than the studies conducted in Jordan (27%) [20], European countries (35%) [21], Malaysia (25%) [22], Taiwan (17%) [23], Bristol city (20%) [24], Vietnam (20.7%) [25], India (32.8%) [26], Iran (21.3%) [27], Tanzania (30.1%) [28] and Addis Ababa (37.8%) [4, 29]. The high level of workplace stress in this study might be due to the study setting, the tools used the, time difference and the study population.

The current study revealed that workplace stress was associated with working experience. This finding is similar with the previous studies done in Mekelle [30]. Similarly, the current study revealed that health care professionals’ educational status was significantly affecting workplace stress which was contradicted with a study done in Saudi Arabia [31] that did not affect workplace stress.

### Conclusion

The current study concluded that the prevalence of workplace stress was high among the study participants. Educational status and working experience were found to be associated with workplace stress. Therefore, this study recommended that the health care facilities should work to identify other sources of workplace stress and further large-scale researches need to be done.

## Limitation

The cross-sectional nature of study design used that suffers from recall bias. The cross-sectional nature of this study did not clearly identified cause–effect relationship of independent variables and dependent variable. The current study was subjective to self-response bias.

## Abbreviations

CEOs: Chief Executive Officers
ERC: Ethical Review Committee
SPSS: Statistical Package for Social Sciences

## References

[1] Pisljar T, van der Lippe T, den Dulk L (2011). Health among hospital employees in Europe: a cross-national study of the impact of work stress and work control. Soc Sci Med.

[2] (ILO) Ilo. Workplace stress: a collective challenge; World Day for Safety and Health at Work 28 April 2016. 2016.

[3] Parent-Thirion A (2007). Fourth European working conditions survey.

[4] Salilih SZ, Abajobir AA (2016). Work-related stress and associated factors among nurses working in public hospitals of Addis Ababa, Ethiopia: a cross-sectional study. Workplace Health Saf.

[5] Rose C, Nabirye KCB, Pryor ER (2011). Occupational stress, job satisfaction and job performance among hospital nurses in Kampala, Uganda. J Nurs Manag.

[6] Bartram T, Joiner TA, Stanton P (2004). Factors affecting the job stress and job satisfaction of Australian nurses: implications for recruitment and retention. J contemp Nurse.

[7] Boran A, Shawaheen M, Khader Y, Amarin Z, Hill Rice V (2011). Work-related stress among health professionals in northern Jordan. Occup Med.

[8] Dowell AC, Hamilton S, McLeod DK (2000). Job satisfaction, psychological morbidity and job stress among New Zealand general practitioners. N Z Med J.

[9] Anand S, Mejid A (2018). Prevalence and associated factors of work related stress among nurses working in worabe comprehensive and specialized hospital, south west Ethiopia. Prevalence.

[10] Gorgich EAC, Zare S, Ghoreishinia G, Barfroshan S, Arbabisarjou A, Yoosefian N (2017). Job stress and mental health among nursing staff of educational hospitals in South East Iran. Thrita.

[11] Bahir Dar City HD Rport R. Bahir Dar Special Zone Health Department human Resource office 2009 physical year report. 2009 E.C.

[12] Cousins R, Mackay CJ, Clarke SD, Kelly C, Kelly PJ, McCaig RH (2004). ‘Management standards’ work-related stress in the UK: Practical development. Work Stress.

[13] Dagget T, Molla A, Belachew T (2016). Job-related stress among nurses working in Jimma Zone public hospitals, South West Ethiopia: a cross-sectional study. BMC Nurs..

[14] Phillips S, Sen D, McNamee R (2007). Prevalence and causes of self-reported work-related stress in head teachers. Occup Med.

[15] Godifay G, Worku W, Kebede G, Tafese A (2018). Work related stress among health care workers in Mekelle City Administration Public Hospitals, North Ethiopia. J Health Med Nurs.

[16] Tabby Maphangela M-D. Factors associated with occupational stress among nurses working in clinics in Gaborone, Botswana. 2015.

[17] Salam A, editor. Job stress and job satisfaction among health care professionals. In: Qatar Foundation annual research conference proceedings. HBKU Press Qatar; 2016.

[18] Kane PP (2009). Stress causing psychosomatic illness among nurses. Indian J Occup Environ Med.

[19] Visser MR, Smets EM, Oort FJ, de Haes HC (2003). Stress, satisfaction and burnout among Dutch medical specialists. Can Med Assoc J.

[20] Boran A, Shawaheen M, Khader Y, Amarin Z, Hill Rice V (2012). Work-related stress among health professionals in northern Jordan. Occup Med.

[21] Buckley P (2015). Work related stress, anxiety and depression statistics in Great Britain.

[22] Robat RM. Occupational stress among nurses in the District Hospital and Health Centres of Temerloh, Pahang. Department of Social and Preventive Medicine, Faculty of Medicine; 2008.

[23] Aoki M, Keiwkarnka B, Chompikul J (2011). Job stress among nurses in public hospitals in Ratchaburi province, Thailand. J Public Health Dev.

[24] Smith A, Johal S, Wadsworth E, Smith GD, Peters T (2000). The scale of occupational stress: the Bristol stress and health at work study.

[25] Minh KP (2014). Work-related depression and associated factors in a shoe manufacturing factory in Haiphong City, Vietnam. Int J Occup Med Environ Health.

[26] Saini N, Agrawal S, Bhasin S, Bhatia M, Sharma A (2010). Prevalence of stress among resident doctors working in Medical Colleges of Delhi. Indian J Public Health.

[27] Soori H, Rahimi M, Mohseni H. Occupational stress and work-related unintentional injuries among Iranian car manufacturing workers. 2008. 18720634

[28] Mkumbo K (2014). Prevalence of and factors associated with work stress in Academia in Tanzania. Int J High Educ.

[29] Salilih SZ, Abajobir AA (2014). Work-related stress and associated factors among nurses working in public hospitals of Addis Ababa, Ethiopia: a cross-sectional study. Workplace Health Saf.

[30] Nyssen AS, Hansez I, Baele P, Lamy M, De Keyser V (2003). Occupational stress and burnout in anaesthesia. Br J Anaesth.

[31] Salam A, Abu-Helalah M, Jorissen SL, Niaz K, Mansour A, Al Qarni A. Job stress and job satisfaction among health care professionals. Eur Sci J. 2014;10(32).

